# Photothermal cellular stimulation in functional bio-polymer interfaces

**DOI:** 10.1038/srep08911

**Published:** 2015-03-10

**Authors:** Nicola Martino, Paul Feyen, Matteo Porro, Caterina Bossio, Elena Zucchetti, Diego Ghezzi, Fabio Benfenati, Guglielmo Lanzani, Maria Rosa Antognazza

**Affiliations:** 1 Center for Nano Science and Technology, Istituto Italiano di Tecnologia, Via Pascoli 70/3, 20133 Milano, Italy; 2 Politecnico di Milano, Dip.to di Fisica, P.zza L. Da Vinci 32, 20133 Milano, Italy; 3 Center for Synaptic Neuroscience, Department of Neuroscience and Brain Technologies, Istituto Italiano di Tecnologia, Via Morego 30, 16163 Genova, Italy; 4 Department of Experimental Medicine, University of Genova, Viale Benedetto XV 3, 16132 Genova, Italy; 5 Politecnico di Milano, Dip.to di Matematica, P.zza L. Da Vinci 32, 20133 Milano, Italy

## Abstract

Hybrid interfaces between organic semiconductors and living tissues represent a new tool for *in-vitro* and *in-vivo* applications, bearing a huge potential, from basic researches to clinical applications. In particular, light sensitive conjugated polymers can be exploited as a new approach for optical modulation of cellular activity. In this work we focus on light-induced changes in the membrane potential of Human Embryonic Kidney (HEK-293) cells grown on top of a poly(3-hexylthiophene) (P3HT) thin film. On top of a capacitive charging of the polymer interface, we identify and fully characterize two concomitant mechanisms, leading to membrane depolarization and hyperpolarisation, both mediated by a thermal effect. Our results can be usefully exploited in the creation of a new platform for light-controlled cell manipulation, with possible applications in neuroscience and medicine.

The ability to selectively control and manipulate the electrical activity of living cells, both *in-vitro* and *in-vivo*, has attracted considerable technological and scientific efforts. Indeed, the availability of new techniques for cell stimulation with high selectivity has allowed gaining important knowledge in the information processing in neural circuits^1^. Besides electrical stimulation, optical tools have been exploited as useful, complementary techniques^2^. In general, their most striking advantage is the possibility to achieve unprecedented spatial and temporal resolution. There are many different options for optically driven cell control, which can be grouped in two major categories: use of photoactive mediators, naturally or artificially expressed in cells (*e.g.*, endogenous or genetically expressed light-sensitive proteins), and use of external photoactive materials placed in the close proximity of the cell and able to convert light into an electrical, mechanical, chemical, or thermal stimulus^3^,^4^. In this second category, research for new functional materials and device architectures obviously plays the key role. Among available materials, conjugated polymers can be considered ideal candidates^5^,^6^: they recently emerged as promising tools for the realization of functional interfaces with living tissues^7^,^8^, to control bioelectrical signal *in-vitro*^9^,^10^,^11^ and for targeted biomedical applications *in-vivo*^12^,^13^, demonstrating in some cases better performances than their inorganic counterparts in terms of biocompatibility, mechanical properties, suitability for deep brain stimulation/recording, sensitivity, contact impedance levels and overall signal-to-noise ratio^10^,^12^. In addition, intrinsic sensitivity to visible light makes them the most obvious choice for optical cell stimulation, as recently demonstrated in different biological preparations, including rat hippocampal neurons^14^, rat neocortical astrocytes^15^ and blind explanted retinas^16^,^17^. In these reports, it was hypothesised that the excitation of cell activity was due to a photo-capacitive charging of the active material surface, but the whole coupling mechanism with the cell was not experimentally elucidated.

Another strategy for cellular photostimulation that has been lately attracting interest is Infrared Neural Stimulation (INS). First proposed in 2005 by Wells *et al.*^18^, it is based on direct illumination with an infrared laser, which is absorbed by water causing local heating^19^. This technique has been proven effective on different systems, from cell cultures^20^,^21^ to *in vivo* modulation of neural activity^18^,^22^,^23^ and pacing of a quail embryo's heart^24^. Interestingly, temperature variations induced either by direct light absorption by water or through the presence of external absorbers, have been shown to induce, depending on the stimulation protocol, both excitation^25^,^26^ and inhibition^23^,^27^ of neural activity. It is therefore of primary importance to elucidate the role of thermal effects also in optical stimulation mediated by organic semiconductors. Here, we study the photo-modulation of the membrane potential in non-excitable cells grown onto conjugated polymer films. As a valuable experimental model, we selected HEK-293 (Human Embryonic Kidney 293) cells, since they allowed to isolate the direct effect of photostimulation as a variation in the plasma membrane potential and to reduce the errors introduced by space-clamp artefacts in the measurements, due to their small size with minimal processes^28^. Besides assessing the presence of a capacitive charging of the polymer/electrolyte interface, we focus here on a concomitant response, independent of the charge generation capability of the active material. We ascribe it to a local heating of the cellular environment upon illumination of the semiconducting polymer; in particular we observe that the temperature increase affects both the membrane capacitance and the ion channels conductance, leading, on different time scales, to depolarizing and hyperpolarizing effects, respectively.

## Results

### Device structure and cell viability assays

**Figure 1:**
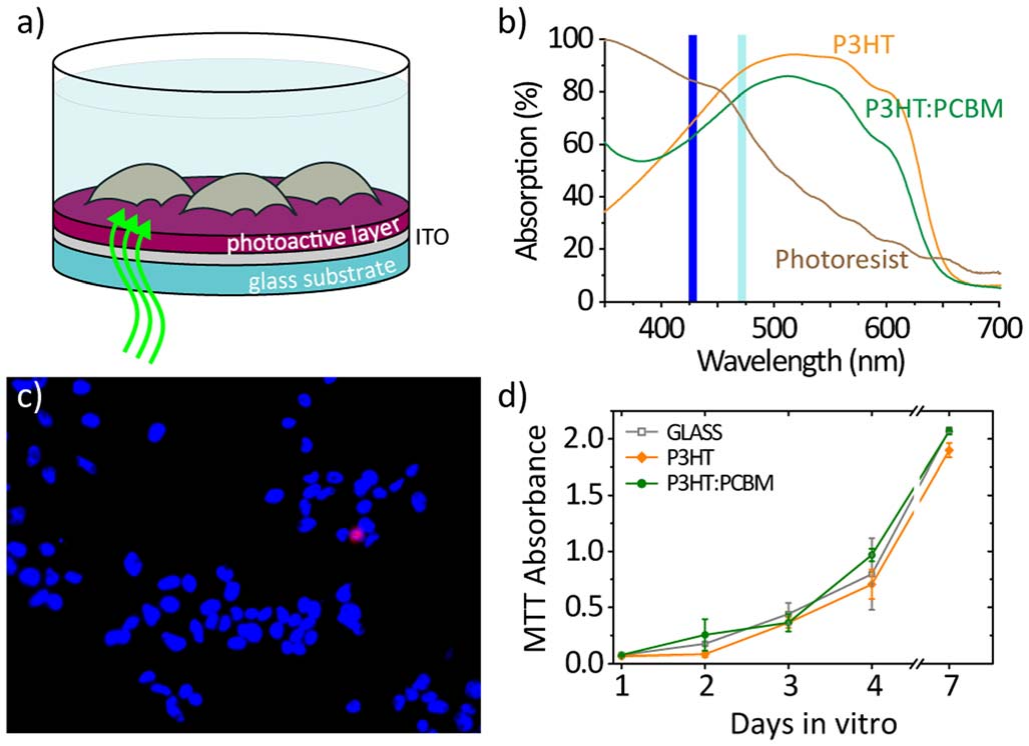
Photoactive polymeric interfaces. (a) Schematic representation of the photoactive interface used in this study. The photoactive layers are spin-casted thin films of P3HT, P3HT:PCBM or a photoresist. The substrate is a glass coverslip, in some cases covered with a conductive ITO layer. (b) Absorption spectra of the different active layer used in the study. At the wavelength used for excitation (435 nm for the photoresist, blue bar; 475 nm for P3HT and P3HT:PCBM, cyan bar) all samples absorb about 80–90% of the incident light. (c) Fluorescence imaging of HEK-293 cells cultured on a P3HT:PCBM substrate and stained with DAPI (blue) and PI (red). (d) MTT assay for cell proliferation up to 7 days *in-vitro* for different substrates.

The devices used for cellular photostimulation consist of an optically absorbing thin layer of about 100 nm thickness deposited on a glass substrate (Fig. 1a) with or without a conductive layer of Indium-Tin Oxide (ITO) in between. The devices were thermally sterilized (120°C for two hours) and treated with fibronectin to promote cell adhesion^29^; cells were then grown directly on the device surface. Three distinct light absorbing materials were used: a semiconducting polymer, poly(3-hexylthiophene) (P3HT), a blend of this polymer with the electron acceptor phenyl-C61-butyric-acid-methyl ester (P3HT:PCBM), and a photoresist (MicroPosit® S1813®). Absorption spectra are shown in Figure 1b. The film thickness was carefully controlled in order to have comparable optical density in all samples (P3HT and P3HT:PCBM devices were excited at λ = 475 nm, the photoresist at λ = 435 nm). The photophysics of the three absorbing layers, however, is quite different. Optical absorption in the photovoltaic blend results in the generation of charge carriers that can escape geminate recombination. Conversely, in the neat polymer, photogenerated charge pairs are fairly localized and they mainly recombine geminately without displacement. Finally, absorption in the photoresist is expected to create neutral states that decay non-radiatively.

Due to energy levels alignment at the interface, the ITO electrode can collect electrons generated in the active layer, thus promoting the formation of a photopotential between the ITO and the counter-electrode in the bath^30^,^31^. This photopotential leads to a capacitive charging of the polymer/electrolyte interface, with the development of a transient potential in the close proximity of the surface (see Supplementary Figure S1). In contrast, in absence of the ITO electrode, charges generated in the active material are not efficiently dissociated and accumulated, and thus recombine without establishing a sizable surface potential at the interface with the extracellular medium.

HEK-293 cells are cultured at 37°C and 5% CO_2_; when at confluence the cells are plated at a density of 15,000 cells/cm^2^ and cultured for 48 h on P3HT, P3HT:PCBM and glass samples. The evaluation of cell viability is performed by staining non-viable cells with propidium iodide (PI) and the total number of cells with 4',6-diamidino-2-phenylindole (DAPI) (Fig. 1c), resulting in viability of HEK-293 cells around 96%, consistent with the data already reported for other types of cells grown on the same polymeric materials^14^,^15^,^29^. The tetrazolium salt (MTT) assay has been used to obtain a quantitative colorimetric evaluation of cell survival and proliferation. We evaluate cell proliferation on P3HT and on P3HT:PCBM at different times (1, 2, 3, 4 and 7 days, *n* = 6 substrates for each point): proliferation increases at increasing incubation times, with similar rates compared to the control glass substrates (Fig. 1d).

### Cell photostimulation on various substrates by short (20 ms) light pulses

**Figure 2:**
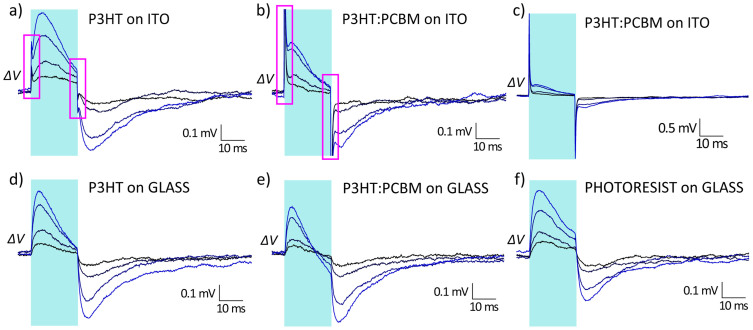
HEK-293 response to photostimulation on different substrates. Membrane potential variation measured in HEK-293 cells cultured on different photoactive substrates under pulsed illumination. Samples on ITO: P3HT (a), P3HT:PCBM (b,c); samples on bare glass: P3HT (d), P3HT:PCBM (e) and photoresist (f). The traces in each panel refer to four increasing light intensities (7.7 mW/mm^2^, 15 mW/mm^2^, 35 mW/mm^2^, 47 mW/mm^2^). Panel (c) is the same measurement as panel (b), but on an extended y-scale. In the magenta boxes of panels (d,e) the fast spikes attributed to the photopotential generation at the polymer/electrolyte interface are highlighted. The cyan shaded areas represent the light pulse duration (20 ms). Each trace is the mean of 25 consecutive sweeps.

The effect of photostimulation of the active substrate on HEK-293 cells was assessed by measuring the light-induced changes in the plasma membrane potential with a standard patch-clamp setup. Whole-cell recordings were carried out in current-clamp (I = 0) configuration in response to 20 ms pulses of light. Representative cellular responses recorded on P3HT and P3HT:PCBM ITO-coated substrates are reported in Fig. 2a,b respectively, for light intensities (λ = 475 nm) ranging from 7.7 to 47 mW/mm^2^. Upon photostimulation, two different signals were observed: a fast positive spike, similar in intensity and temporal dynamics to the capacitive charging observed in polymer samples without cells (Supplementary Figure S1), followed by a slower transient depolarisation of the cell membrane. When the light was switched off, a complementary behaviour was recorded: a fast negative spike occurred at the offset of the light, followed by a transient hyperpolarisation before the membrane returned to its resting state. While the fast spiking activity was clearly more intense in P3HT:PCBM (as can be better appreciated in Fig. 2c, where the full extent of the spike is shown, in close analogy with what observed in the absence of cells, see Figure S1), the slower depolarisation/hyperpolarisation signal intensity was similar for the two samples.

To better understand the origin of these signals, we removed the ITO electrode and deposited the polymers (P3HT and P3HT:PCBM) directly on glass (Figure 2d,e). In both cases, the fast spikes were absent, while a comparable depolarisation/hyperpolarisation signal was still present. Based on these observations, and on the direct comparison with measurements of capacitive charging of the interface (Supplementary Figure S1), we attribute the fast spiking signal to the surface potential that is generated in devices with the ITO contact. Instead, the origin of the slower component of the signal appears to be independent of the electrical processes occurring upon photoexcitation, as also confirmed by measurements performed on photoresist samples (Figure 2f), which do not support the generation of charges.

**Figure 3:**
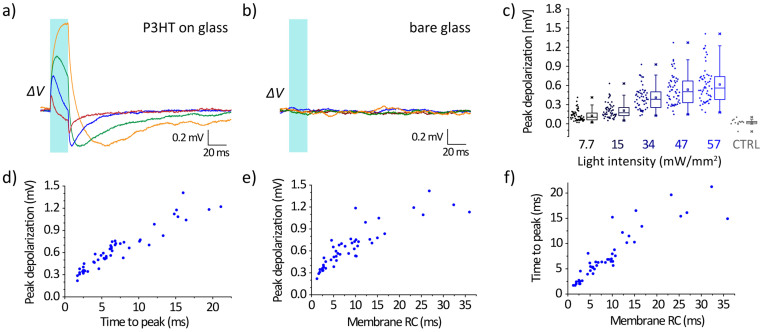
Analysis of cell responses to 20 ms light pulses. (a,b) Membrane potential variation upon illumination (20 ms pulse, 57 mW/mm^2^) in four different HEK-293 cells on P3HT/glass substrates (a); no response could be recorded in control measurements on bare glass substrates (b). (c) Statistical distribution (*n* = 51) of the maximum depolarisation recorded for different intensities of illumination; CTRL refers to the maximum potential variation recorded for cells on the bare substrates (*n* = 12). (d) Correlation (*n* = 48) between the time to peak of the depolarising signals recorded on the P3HT/glass samples and the actual values of depolarisation reached (*r*^2^ = 0.89). (e,f) Correlation (*n* = 48) between peak depolarisation (e, *r*^2^ = 0.75) and time to peak (f, *r*^2^ = 0.80) with the time constant of the membrane equivalent circuit. Points in d–f represent data from individual cells.

Given the qualitative similarity of slow depolarization behavior in all samples reported in Figure 2, we turned to characterize in depth this component of the response utilizing glass/P3HT devices. The responses of four representative HEK-293 cells to 20 ms light pulses (57 mW/mm^2^) are reported in Fig. 3a. As already observed, in all cases cells showed a transient depolarisation of the membrane potential upon illumination, which turns into hyperpolarisation when light is turned off. This response is due to light absorption by the active material, since it cannot be ascribed neither to endogenous chromophores present in the cells nor to absorption by water^19^,^32^, as demonstrated by the absence of any effect in HEK-293 cells cultured on glass substrates without the polymer (Fig. 3b). It is also evident that the response obtained upon photostimulation had a large variability from cell to cell, as it clearly emerges from the boxplots in Fig. 3c, where the peak depolarisations for various illumination intensities are reported (*n* = 51 cells). However, a clear correlation between the maximum depolarisation amplitude and the time to reach it was observed (Fig. 3d). This variability can be attributed to the intrinsic variability of the electrical parameters of the investigated cells as a clear correlation of both maximum depolarisation amplitude (Fig. 3e) and time to peak (Fig. 3f) with the characteristic time constant of the membrane equivalent circuit (*i.e.* the product of membrane resistance and capacitance) was observed.

### Cell photostimulation with long (200 ms) light pulses

**Figure 4:**
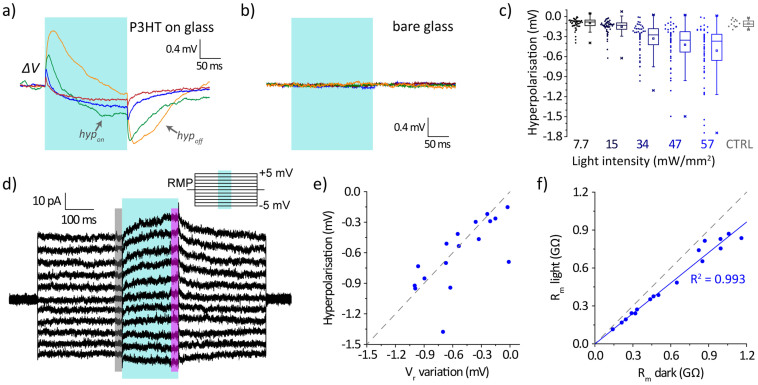
Analysis of cell response to 200 ms light pulses. (a,b) Membrane potential variation upon illumination with long light pulses (200 ms pulse, 57 mW/mm^2^) for the same four HEK-293 cells of Fig. 3, in the case of P3HT/glass substrates (a) or bare glass substrates (b). (c) Statistical distribution (*n* = 51) of the maximum hyperpolarisation during the light pulses; CTRL refers to the maximum (negative) variation recorded for cells on bare substrates (*n* = 12). (d) Membrane response to a voltage-clamp step protocol (holding value at resting membrane potential, RMP, with steps from −5 mV to +5 mV in 1 mV steps) with a 200 ms light pulse stimulation (57 mW/mm^2^, cyan rectangle); the grey and magenta boxes represent the regions from which the membrane characteristics (membrane potential and resistance) were calculated for the dark and light conditions, respectively. (e) Correlation between the cell hyperpolarisation measured in current-clamp experiments and the variation in reversal potential (as measured from the protocol of panel d); the grey dashed line represents the quadrant bisector. (f) Correlation between the variation in cell membrane resistance in the dark and at the end of the light stimulus; the grey dashed line represent the quadrant bisector, while the solid blue line represent the line best fitting the data, with a slope of 0.804 ± 0.017. Points in e,f represent data from individual cells.

The depolarising response upon illumination was transient; in fact, as it can be seen in Fig. 3a, after some time the depolarisation started to decay and was followed by hyperpolarisation. In the case of longer stimuli (200 ms), this depolarisation/hyperpolarisation switch was more evident and occurred while the light stimulus was still on (Fig. 4a). To avoid confusion between the progressive hyperpolarisation observed during the light stimulus and the transient hyperpolarisation occurring when the light is switched off, we will refer to them in the following as “*hyp_on_*” and “*hyp_off_*”, respectively (see Fig. 4a).

In control substrates, without the light absorbing layer, no signal was detected (Fig. 4b). As in the case of the depolarisation signal, the extent of the *hyp_on_* component was variable among individual cells (Fig. 4c); however, in contrast with the previous case, there was no evident correlation between *hyp_on_* amplitude and membrane time constant, nor with other basic electrical properties of the membrane (such as membrane capacitance or resting potential, see Supplementary Figure S2). To get a better insight on this effect, we analyse the membrane electrical characteristics extracting the cell I–V curves near the resting membrane potential (RMP) in the dark and after 200 ms illumination (Fig. 4d). The cell was held at the RMP and a series of voltage steps (from −5 mV to +5 mV, in 1 mV steps) were applied in voltage-clamp configuration, as depicted in the inset of Fig. 4d. During the protocol, the cell was illuminated by a 200 ms, 57 mW/mm^2^ light pulse (light blue box in the inset). From the recorded current traces (Fig. 4d), we extracted the membrane I–V curves in dark (as an average over the 20 ms period before switching on the light) and upon illumination (as an average over the 20 ms period before switching off the light, Supplementary Figure S3). From the I–V curves, the reversal potential and the membrane resistance were then calculated. The reversal potential exhibited a variation towards more negative values ranging from few hundreds of μV up to 1 mV (*n* = 17 cells), which correlates well with the hyperpolarisation measured on the same cell in current-clamp measurements (Fig. 4e). The membrane resistance decreased as well upon illumination, with a variation of about 20% (Fig. 4f).

### Analysis of the thermal response

**Figure 5:**
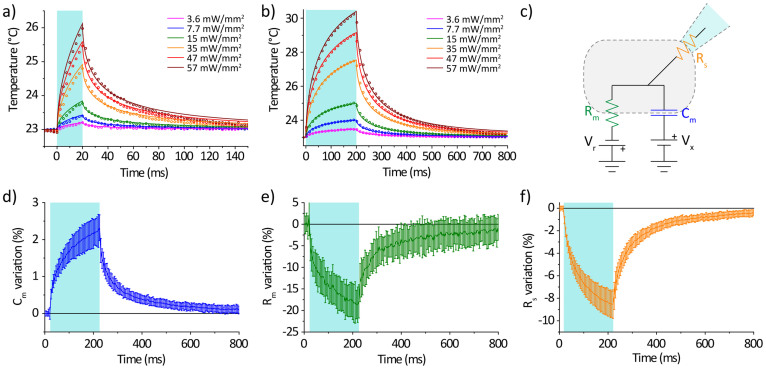
Thermal response of the photoactive interface and its effect on cell membrane. (a,b) Increase in temperature measured (open circles) in the bath in close proximity to the polymer (P3HT on glass) surface for 20 ms (a) and 200 ms (b) pulses at different light intensities. Solid lines represent numerical simulation of the thermal diffusion problem. (c) Equivalent circuit representation of a cell membrane in a patch-clamp measurement. *R_m_* and *C_m_* are the membrane resistance (that includes the effect of all HEK-293 ion channels) and capacitance, respectively, *R_s_* the series resistance of the patch, *V_r_* the reversal potential and *V_x_* a term needed to take into account the asymmetries between inner and outer membrane surface charges and ion distributions. (d,e,f) Time evolution of membrane capacitance (d), membrane resistance (e) and series resistance (f) during illumination (200 ms, 57 mW/mm^2^ – cyan rectangle), measured for *n* = 39 cells (error bars represent standard deviations).

We demonstrated above that the observed changes in membrane potential are independent of charge generation in the light absorbing material. Accordingly, a photo-thermal effect due to the heating of the bath in proximity of the device surface is the most plausible origin of the observed phenomena, similar to what happens with IR neural stimulation upon absorption by water^19^. To corroborate this hypothesis, we measured the bath temperature variation in the close proximity of the absorbing layer by using the method of the calibrated pipette resistance^33^ (Supplementary Figure S4). The temporal profiles of the local heating for 20 ms-long and 200 ms-long pulses are reported in Fig. 5a,b (open circles) for increasing light intensities (see Supplementary Figure S5 for measurements on photoresist samples). We observed that, during illumination at the maximum intensity, the temperature roughly increased by 3°C and 7°C, respectively. Numerical simulations of heat diffusion in the system (solid traces in Fig. 5a,b; for more details see Supplementary Discussion and Supplementary Figure S6) fully supported the experimental data. The increase in temperature is localized to the region where the light is impinging on the absorbing substrate, while the bulk of the bath remains at the baseline temperature during the stimulation, as can be seen from spatial and temporal temperature distribution reported in Supplementary Figure S7.

By applying a sinusoidal voltage-clamp paradigm to the cell (see Supplementary Discussion for more information), modelled with the equivalent circuit of Fig. 5c, we extracted the variations of the membrane capacitance *C_m_*, membrane resistance *R_m_* and series resistance *R_s_* during a 200 ms light pulse (57 mW/mm^2^) (Fig 5d–f, *n* = 39 cells). The traces show that the three parameters closely followed the variation in the local temperature, with the capacitance increasing and the two resistances decreasing. The increase of *C_m_*, during the 200 ms-long light pulse, is about 2%, while the *R_m_* variation of 18 ± 4% is in good agreement with the values reported in Fig. 4f. Regarding *R_s_*, the measured 8–9% decrease at the end of the 200 ms light pulse can be related to a similar decrease in the pipette resistance upon heating (see Supplementary Figure S4).

### Numerical simulations of the membrane potential responses to photostimulation

We then carried out numerical calculations of the cell equivalent circuit (Fig. 5c) in current-clamp configuration (with zero current flowing in *R_s_*) to simulate the variation upon illumination in the cell membrane potential, taking into account the temperature-dependence of the different parameters. In particular, we modelled the following effects of heating on the plasma membrane: (i) the increase in capacitance, which has been already reported as a cause of transient depolarisation in cell potential^20^; (ii) the variation in membrane resistance and the associated decrease in reversal potential, which is expected to be the cause of the *hyp_on_* hyperpolarisation.

Based on the measurements in Fig. 5 and on the existing literature^20^, we assumed that the increase in the membrane capacitance is proportional to the increase in temperature, according to the following relation:
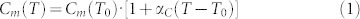
with *α_C_* representing the relative increase for 1°C temperature change and *T_0_* the baseline room temperature. For *R_m_* we assumed a temperature-dependence expressed by the widely used temperature coefficient *Q_10_*:^34^
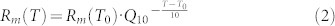
In the following, we assumed a value of *Q_10_* = 1.3, which well reproduces the measured variation of *R_m_* measured in Fig. 5e. However, temperature-induced modifications of ion channel conductivities do not only affect *R_m_*, but also determine a different equilibrium condition for ion transport across the membrane, since different channels are expected to have slightly different temperature coefficients. According to the Goldman-Hodgkin-Katz (GHK) equation, the membrane potential is given by:
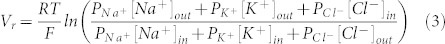
where *R* is the ideal gas constant, *F* is the Faraday constant and *P_X_* the membrane permeability for the ionic species *X*. The implementation of this equation in a numerical simulation is not straightforward, since it relies on the knowledge of the actual ion channel expression in the membrane and their specific temperature coefficients. We thus approximate this dependence with the following empirical power law:
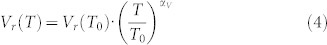
where *α_V_* is a fitting parameter dependent on the ion channel properties of specific cells.

**Figure 6:**
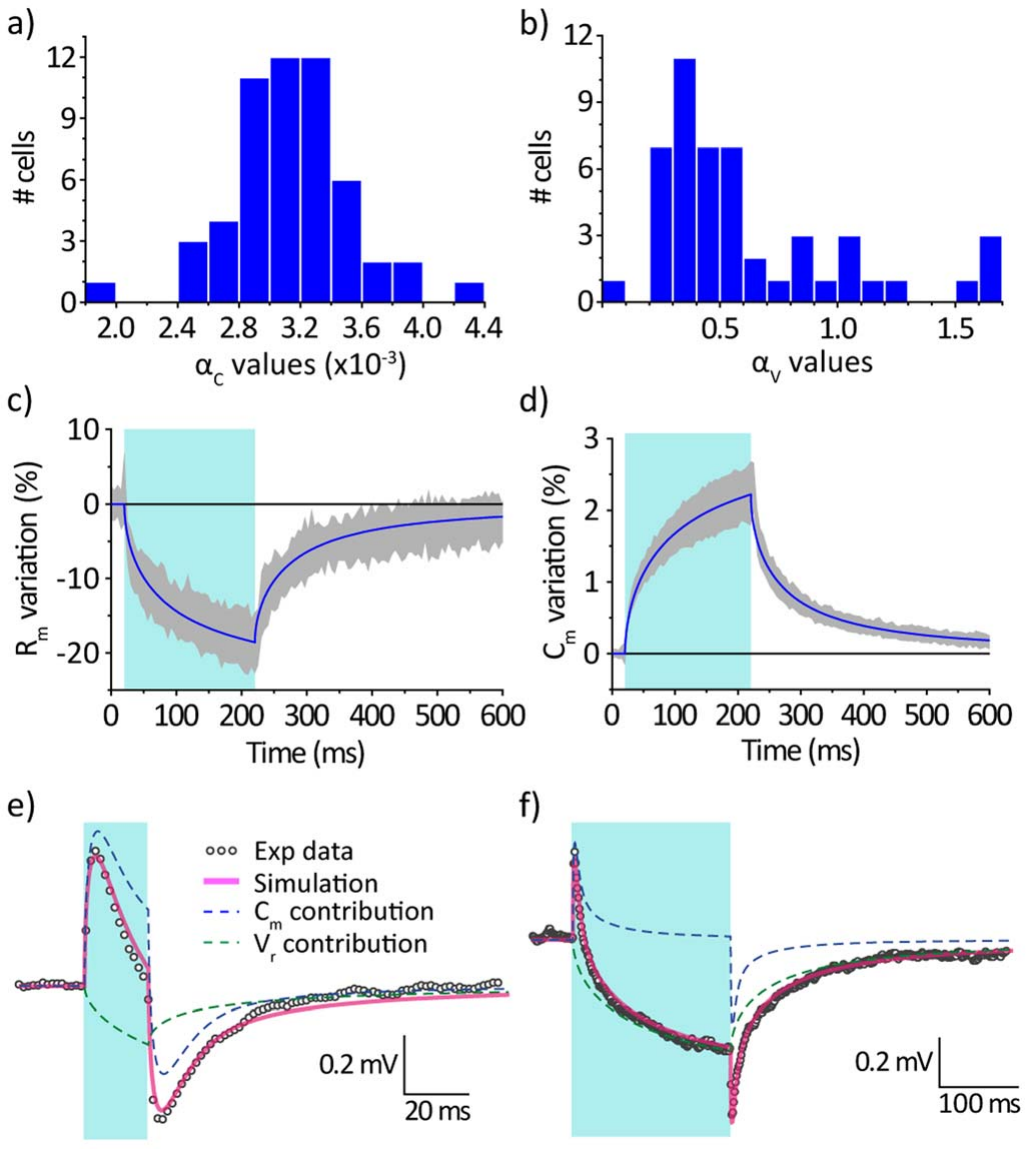
Numerical simulations of the membrane potential responses to photostimulation. (a,b) Statistical distribution for the parameters *α_C_* (a) and *α_V_* (b) of the numerical model obtained from the fitting of the experimental curves (*n* = 54 cells). (c,d) Simulated time evolution (solid blue lines) of the membrane resistance (c) and membrane capacitance (d) for typical values of the model parameters (*α_C_* = 0.0032, *α_V_* = 0.375), compared to the values measured from Fig. 5 (shaded grey regions). (e,f) Comparison between the membrane potential experimental measurements (grey dots) and numerical simulation (magenta lines) for a typical cell under photoexcitation with 20 ms (e) and 200 ms (f) pulses (cyan rectangles). Dashed lines represent the calculated contributions to the final potential traces from the variation in membrane capacitance (blue) and reversal potential (green).

This model has been used to fit current clamp measurements, both with 20 ms- and 200 ms-long light pulses. The values for the free parameters α_C_ and α_V_ have thus been determined and their distribution is reported in Fig. 6a,b. The fitted values for *α_C_* have a Gaussian distribution with mean value 0.0031 K^−1^ and a standard deviation of 0.0004 K^−1^, which is in very good agreement with data reported by Shapiro *et al.*^20^ A different trend is instead observed for the values of α_V_, which show a more pronounced variability.

Typical simulated temporal traces for the membrane resistance and capacitance are reported in Fig. 6c,d respectively, in good agreement with the experimental data (Fig. 5d,e). Finally, the simulated traces of the membrane potential are reported for a typical cell in Fig. 6e,f for both 20 ms- and 200 ms- light pulses (magenta solid lines); the agreement with experimental data (black symbols) is satisfactory. The relative contributions to the overall signal of the two considered mechanisms, *i.e.* the change in capacitance and the shift in the reversal potential, are also highlighted (blue and green dashed lines). They clearly show that the transient depolarisation and hyperpolarisation *hyp_off_* are related to the fast changes in membrane capacitance, while the hyperpolarisation *hyp_on_* is due to the variation of the reversal potential following the temperature increase.

## Discussion

Results presented above provide a detailed picture of the Cell Stimulation by Polymer photoexcitation (CSP) mechanism in HEK-293 cells. We find that two distinct mechanisms participate in cell stimulation upon illumination: a capacitive charging of the polymer/electrolyte interface and a local heating of the material.

The capacitive charging of the interface requires the presence of an ITO electrode in order to collect electrons generated in the active material upon illumination and promote the formation of a photo-potential. The intensity of this signal depends on the charge generation yield in the absorbing material: it is high for the photovoltaic P3HT:PCBM blend, low for the pristine P3HT polymer and null for the photoresist layer. Capacitive stimulation of neurons has been already demonstrated by various groups with different architectures, both with purely electrical devices^35^ and through optical excitation^17^,^36^. Interestingly, previous reports have demonstrated that such a mechanism is able to elicit the response of exogenous voltage-gated channels specifically expressed in HEK-293 cells^37^,^38^. However, since in our case non-transfected HEK-293 cells were used, we do not expect a sizable response from the few endogenous channels present in the cell.

The second phenomenon identified in our device is the thermal heating of the bath at the interface with the active layer. Light absorption by the polymer leads to the generation of different photoexcited states in the material. Given that we are not extracting photocurrent from the device and the low photoluminescence yield, excited states will recombine non-radiatively to the ground state, thus releasing thermal energy. Indeed, an increase in the temperature of the extracellular space has been measured upon illumination, with incremental values up to 7°C for 200 ms pulse at the maximum intensity used (57 mW/mm^2^).

Heating of the extracellular bath has a clear effect on the cell membrane electrical properties, in terms of capacitance, resistance and resting potential. We could experimentally measure an increase in cell capacitance of about 0.3% for each degree of temperature increase. Since the charge stored on the cell membrane cannot change instantaneously, at short times after the onset of the illumination this variation of capacitance leads to a variation in membrane potential given by:
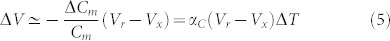
which has been derived assuming a capacitor with constant stored charge in the approximation of small signals. Here *C_m_* is the membrane capacitance, *V_r_* is the membrane reversal potential and *V_x_* a term that takes into account the asymmetry of charging on the two sides of the plasma membrane (see circuit in Fig. 5c). Based on our numerical simulations, a value of *V_x_* = 160 mV has been estimated, consistent with the range of values reported in literature for the same cells^20^, while *V_r_* is usually around −30 mV for HEK-293 cells.

At later times, charges will start to flow across the membrane to counterbalance this change in capacitance and restore the equilibrium potential, on a timescale determined by the membrane time constant (*i.e.* the product *τ = R_m_C_m_*). The actual value of the depolarisation reached is thus resulting from the competition between the time constant *τ* and the slope of the temperature increase. These observations are consistent with the data reported in Fig. 3 showing the transient depolarisation measured upon illumination. Cells with a more resistive membrane (and thus a larger time constant) have slower and more intense responses, while more conductive cells present a very low and fast depolarisation signal. Variation of the plasma membrane capacitance has been recently suggested as the main mechanism for the depolarisation observed under thermal stimulation in different types of cells^20^. The underlying phenomenon could reside either on electrostatic origin, namely a variation in the size of the diffuse layers at the two sides of the plasma membrane, as proposed by Shapiro *et al.*, or on a temperature-dependent modification of the physical properties of the membrane lipid bilayer. Cell membranes, under physiological conditions, are close to phase transition^39^; a slight temperature increase might easily lead to a different order in the gel or liquid phase of the membrane, thus implying a reduction in its thickness and an overall increase in the capacitance.

The increase in temperature also affects the conductance of the ion channels present in the cell membrane, leading to two different effects: a decrease of the membrane resistance and of the cell reversal potential. The effect on membrane resistance is due to the fact that ion transport through membrane channels is enhanced with increasing temperature. In particular, from the measurement of Fig. 5e we estimated a temperature coefficient (*Q_10_*) for the process of roughly 1.3, which is in the range of literature data for many different ion channels^40^. The variation in the reversal potential (*V_r_*) is explained by the GHK equation (Eq. 3), where it depends directly on temperature in the prefactor, resulting in a gradual hyperpolarisation of the cell at increasing temperature. This dependence is, at least qualitatively, consistent with our experimental observation for the longer stimuli (Fig. 4). However, there is also implicit dependence on the permeability coefficients *P_X_*. The logarithmic part of the GHK equation will thus have a non-trivial temperature dependence, based on the expression of ion channels in each cell and their different temperature coefficients, that we simplified using the empirical relation of Eq. 4.

The numerical simulations carried out demonstrate that the phenomena discussed above fully account for the thermal component of the CSP mechanism in HEK-293 cells, explaining both the transient depolarisation observed at short times and the hyperpolarisation for longer stimuli as a consequence of local heating of the cell membrane.

Interestingly, recent reports have shown that, while a brief (in the millisecond range) and intense temperature increase is able to elicit action potential firing^25^,^26^, local heating on longer timescales results in an inhibition of neural activity^23^,^27^. These observations are consistent with our results of a biphasic behaviour of depolarisation-hyperpolarisation in the cell membrane potential.

Translating the findings presented here to more complex systems, like neurons, is not straightforward, but is a valuable starting point to understand the different mechanisms that may take part in neural stimulation. The light intensities used in this work are comparable to the ones used in our previous reports in neurons^14^; however considering the depolarisation amplitudes measured in the present work, thermal-induced variation of membrane capacitance cannot be invoked as the sole excitation mechanism. Therefore, we should consider that, in more complex systems, such as neurons or explanted retinas, other photo-transduction mechanisms are present, sensitive both to thermal^26^ and electrical stimuli^37^,^38^. A capacitive charging of the interface could play a role as also reported in other works with similar architectures^17^,^36^. Moreover, beside a variation in membrane capacitance, other temperature-related mechanisms can occur in neural systems; for example, local heating has been demonstrated to trigger the opening of temperature-sensitive ion channels and subsequent firing of action potentials, as demonstrated for IR laser stimulation in sensory neurons^26^.

Light-mediated thermal stimulation of several different cells and tissues, both *in-vitro*^20^,^21^ and *in-vivo*^22^,^23^, has already been reported in literature, but in these studies water absorption of an IR laser beam was used as excitation. With respect to such systems, the CSP mechanism has distinct advantages. It is based on light of moderate intensity and in the visible range, which can be provided by standard fluorescence microscopy set-ups, while water absorption requires wavelengths in the IR (mainly ~1.45 μm and ~1.93 μm), which are not compatible with the optical systems of standard microscopes. For this reason IR stimulation is usually delivered to the preparation by external sources via optical fibres, which have to be mechanically positioned and their output cannot be easily focused in a small spot. On the contrary, using spatial light modulators or laser scanning systems directly coupled to the microscope optical train, photoactive substrates can allow independent stimulation of multiple cells with enhanced temporal and spatial resolution, since visible light can be easily focused down to diffraction-limited spots by the microscope objective.

The CSP process can be developed as a new, complementary tool in neuroscience. CSP offers a number of potential advantages, like the low invasiveness, the selectivity in space and time, the high biocompatibility, the possibility to be easily coupled to any existing electrophysiological working station, without requiring complex techniques or dedicated set-ups for optical stimulation. We believe that the photoexcitation of living cells mediated by polymer absorption is a new tool that can be usefully exploited in electrophysiology and can be developed into a platform for cell control by light with applications in neuroscience and medicine.

## Methods

### Sample preparation

Regioregular P3HT (99.995% purity, M_n_ 54.000–75.000 molecular weight) was purchased from Sigma Aldrich; PCBM (99.5% purity) was purchased from Nano-C; Microposit® S1813 photoresist was purchased from Shipley. All materials were used without any further purification. The samples for cell cultures and surface potential measurements were prepared by spin-coating on square 18×18 mm^2^ glass (VWR) or glass/ITO (10 Ω/sq, from NanoCS) substrates, carefully rinsed in successive ultrasonic baths of nanopure water, acetone and isopropanol. P3HT and P3HT:PCBM (1:1 wt) solutions were prepared in chlorobenzene at a final P3HT concentration of 20 g/l. They were spin-coated on the cleaned substrates with a two-steps recipe: i) 3 s at 800 rpm, ii) 60 s at 1600 rpm. All films were thermally treated in an oven at 120°C for 2 h for annealing and sterilization. To promote adhesion, samples for cell cultures were coated with fibronectin (from bovine plasma, Sigma Aldrich) at a concentration of 2 μg/ml in phosphate buffered saline (PBS) for at least 30 min at 37°C and then rinsed with PBS.

### Cell culture maintenance

All procedures were performed using immortalized cell lines, in accordance with the principle of the 3R (Replacement, Reduction, Refinement) as established by the European Community Council (Directive 2012/63/EU of 22 September 2010) and were approved by the Italian Ministry of Health.

HEK-293 cells were cultured in cell culture flasks containing Dulbecco's modified Eagle's medium (DMEM) added with 10% Fetal Bovine Serum (FBS), 100 U/ml Penicillin and 100 μg/ml Streptomycin. Culture flasks were maintained in a humidified incubator at 37°C with 5% CO_2_. When at confluence, HEK-293 cells were enzymatically dispersed using trypsin-EDTA and then plated on our substrates at a concentration of 15,000 cells/cm^2^.

### Viability Assay

Cells were stained *in-vivo* with propidium iodide (PI), fixed in 4% paraformaldehyde and 4% sucrose under dark conditions for 20 min, and subsequently stained with 4',6-diamidino-2-phenylindole (DAPI) to reveal cell nuclei. Images were acquired using a fluorescence microscope, and PI/DAPI cell counting ratio was calculated using the Image J software.

### MTT assay

P3HT, P3HT:PCBM and glass samples were prepared as described above. HEK-293 were plated on each substrate at a concentration of 15,000 cells/cm^2^. Proliferation was evaluated after 1, 2, 3, 4 and 7 days with the MTT assay two in replicates. For each time point, the culture medium was removed and replaced with fresh medium without serum and phenol red, supplemented with 0.5 mg/ml of MTT reagent; cells were re-incubated at 37°C for 2 h. Culture medium was then removed and 1 ml of ethanol was added to dissolve formazan crystals. The absorbance of the solution (at 560 nm) was measured with a spectrophotometer (Cary 50, Agilent Technologies).

### Electrophysiology

Intracellular recordings were performed using a patch-clamp setup (Axopatch 200B, Axon Instruments) coupled to an inverted microscope (Nikon Eclipse Ti). HEK-293 cells (laboratory passage 20–22) were measured at 1–3 DIV in whole-cell configuration with freshly pulled glass pipettes (3–6 MΩ), filled with the following intracellular solution [mM]: 12 KCl, 125 K-Gluconate, 1 MgCl_2_, 0.1 CaCl_2_, 10 EGTA, 10 HEPES, 10 ATP-Na_2_. The extracellular solution contained [mM]: 135 NaCl, 5.4 KCl, 5 HEPES, 10 Glucose, 1.8 CaCl_2_, 1 MgCl_2_. Only single HEK-293 cells were selected for recordings. All measurements were performed at room temperature. Acquisition was performed with pClamp-10 software (Axon Instruments) and data were analyzed with Matlab (Mathworks).

### Optical excitation

The light source for excitation of the polymer was provided by a LED system (Lumencor Spectra X) fibre-coupled to the fluorescence port of the microscope; the illuminated spot on the sample had an area of 0.23 mm^2^. Light powers of the cyan LED used (central wavelength λ = 475 nm) ranged from about 0.8 to 13 mW, measured at the output of the microscope objective (*P_obj_*). Due to optical losses in passing the petri-dish and the substrate, and especially due to the high absorption from the active material, the actual optical power reaching the cells layer, for the maximum case of *P_obj_* = 13 mW (corresponding to an impinging intensity of 57 mW/mm^2^), was reduced to about *P_cell_* = 0.39 mW (which correspond to an intensity of 1.7 mW/mm^2^). As for the deposited energy, in the case of 20 ms pulses, the maximum pulse energy density impinging the sample can be calculated as 1.14 mJ/mm^2^, corresponding to 34 μJ/mm^2^ actually reaching the cells.

## Author Contributions

N.M. performed the opto-electronic characterisation of the substrates, the electrophysiology measurements, analyzed the data and wrote the manuscript. M.P. performed all numerical simulation. C.B. prepared cell cultures and performed vitality measurements. E.Z. performed measurements on photoresist. D.G. and P.F. helped in electrophysiology measurements and interpreted the data. F.B. and G.L. planned the experiments and supported the research. M.R.A. planned the experiments, interpreted the data and wrote the manuscript. All authors discussed the results and revised the manuscript.

## Supplementary Material

Supplementary Information Supplementary info
