# Supplementary material for: Photothermal cellular stimulation in functional bio-polymer interfaces
Source: Sci Rep. 2015 Mar 10;5:8911. doi: 10.1038/srep08911 (PMC4354102; doi:10.1038/srep08911)
Supplement: Supplementary Information — Supplementary info [file srep08911-s1.pdf]

Supplementary Information

Photothermal cellular stimulation in functional bio-polymer interfaces

Nicola Martino[1,2], Paul Feyen[3,4], Matteo Porro[1,5], Caterina Bossio[2], Elena Zucchetti[2], Diego Ghezzi[3], Fabio Benfenati[3,4], Guglielmo Lanzani[1,2,*], Maria Rosa Antognazza[1,*]

[1] Center for Nano Science and Technology, Istituto Italiano di Tecnologia, Via Pascoli 70/3, 20133 Milano, Italy

[2] Politecnico di Milano, Dip.to di Fisica, P.zza L. Da Vinci 32, 20133 Milano, Italy

[3] Center for Synaptic Neuroscience, Department of Neuroscience and Brain Technologies, Istituto Italiano di Tecnologia, Via Morego 30, 16163 Genova, Italy

[4] Department of Experimental Medicine, University of Genova, Viale Benedetto XV 3, 16132 Genova, Italy

[5] Politecnico di Milano, Dip.to di Matematica, P.zza L. Da Vinci 32, 20133 Milano, Italy

[*] guglielmo.lanzani@iit.it; [*] mariarosa.antognazza@iit.it

Supplementary Discussion

Numerical simulations of thermal diffusion

Heat diffusion in the device has been modelled using standard heat diffusion theory and assuming that all the energy absorbed by the polymer is transferred to the system as heat, meaning that the stored electrostatic energy is negligible. A section of the considered tri-dimensional computational domain is reported in Supplementary Figure S5. The problem to be solved is

$$c_p \rho \frac{\partial T}{\partial t} - \nabla \cdot k \nabla T = G(x,t) \qquad \text{in } \Omega_{pol}^{light} \qquad (1a)$$

$$c_p \rho \frac{\partial T}{\partial t} - \nabla \cdot k \nabla T = 0 \qquad \text{in } \Omega \setminus \Omega_{pol}^{dark} \qquad (1b)$$

$$k \nabla T \cdot \nu = 0 \qquad \text{on } \Gamma_{sub} \qquad (1c)$$

$$T = T_{eq} \qquad \text{on } \Gamma_{eq} \qquad (1d)$$

$$T(x,0) = T_{eq} \qquad \text{in } \Omega \qquad (1e)$$

The right hand side terms of (1a) and (1b) reflect the fact that heat is generated only in the illuminated region of the polymer. The rate *G(x,t)* follows the exponential absorption profile following the Beer-Lambert law

$$G(x,t) = \begin{cases} \alpha_{P3HT} I \exp(-\alpha_{P3HT} d(x)) & t \leq t < t_{light} \\ 0 & \text{otherwise} \end{cases} \qquad (2)$$

α_{P3HT} is the polymer absorption coefficient, *I* the light intensity and *d(x)* the distance of point *x* from the incidence plane. Boundary condition (1c) indicates that no heat flows from the device to the plastic substrate, while (1d) denotes the fact that the computational domain is chosen to be wide enough to consider the solution on the boundary Γ_{eq} to be unperturbed, with the temperature equal to the initial value T_{eq}.

Model parameters are listed in the following Table:

	water	P3HT	glass
c_p [J/(Kg·K)]	$4.1813 \cdot 10^3$	$1.4 \cdot 10^3$ [1]	$0.84 \cdot 10^3$
ρ [Kg/m^3]	1000	1100 [2]	2500
k [W/(m·K)]	0.6	0.2 [3]	1.0
α [cm^{-1}]		10^5	

Spatial discretisation of system (1) is performed using the exponentially fitted Galerkin finite element method studied in [4] while time advancing is treated using adaptive BDF formulas [5]. The implementation has been carried out using the open source software Octave [6].

The computed values we show in the main manuscript (Fig. 5a,b) are those attained by the numerical solution $T(x,t)$ in proximity of the solution/polymer interface at the centre of the spotlight.

Extraction of cell membrane electrical parameters

To measure the variation in time of the membrane electrical parameters during a 200 ms-long pulse of light, we analyzed the cell response to an oscillating voltage input. The cell, measured in voltage-clamp mode, was hold at its resting potential and on top of it the following perturbation was applied:

$$V = A\big(sin(2\pi f t) + sin(4\pi f t)\big)$$

with A = 10 mV and f = 195.3125 Hz (corresponding to a period of T = 5.12 ms). In complex notation, with $\omega = 2\pi f$, it can be written as:

$$\tilde{V} = \tilde{V}_{\omega} + \tilde{V}_{2\omega}, \qquad with\ \tilde{V}_{\omega} = Ae^{i\omega t - i\pi/2}$$

The system complex impedance, from the equivalent circuit of Fig. 5c of the main text, is given by:

$$\tilde{Z}_{\omega} = \frac{R_s + R_m + i\omega C_m R_s R_m}{1 + i\omega C_m R_m}$$

The expected current is then given by:

$$I = Re[\tilde{I}], \qquad with\ \tilde{I} = \frac{\tilde{V}_{\omega}}{\tilde{Z}_{\omega}} + \frac{\tilde{V}_{2\omega}}{\tilde{Z}_{2\omega}}$$

We separately considered single periods of 5.12 ms of the measured currents and we fitted the experimental values by using the model presented above. The instantaneous values of R_s, R_m and C_m, where thus calculated every 5.12 ms, and the time traces of Fig. 5d-f were finally reconstructed.

Numerical simulations of cell potential

We modelled the cell membrane with the *RC* circuit represented in Fig. 5c of the main manuscript; considering that the membrane potential measurements are performed in current clamp configuration (*i.e.* no current flowing through R_s), we can write the following Kirchhoff's law

$$\frac{\partial}{\partial t}[C_m(V - V_x)] + \frac{V - V_r}{R_m} = 0.$$

where V is the cell potential with respect to ground. The time evolution of the different electrical parameters is dictated by the constitutive relations for C_m, R_m and V_r that have been presented in the main manuscript (Eq. 1, 2, 4). The temperature dynamics $T(t)$ under illumination were obtained from the heat diffusion numerical simulations, which well represent the experimental measurements. The problem has been numerically solved with Matlab, to obtain the time evolution of the membrane voltage V; for the time advancement method we opted for the function ode15i.

For each considered cell, the corresponding patch clamp measurements were fitted for both stimulation protocols (20 ms-long and 200 ms-long pulses). Considered fitting outputs were R_m, C_m and V. The best fits were obtained by fixing the value of V_x to 160 mV, consistently with values reported in ref. [7]. Fitting procedure allowed to obtain fully satisfactory agreement with experimental values shown in Fig. 5d and 5e in the main manuscript.

Supplementary Figures

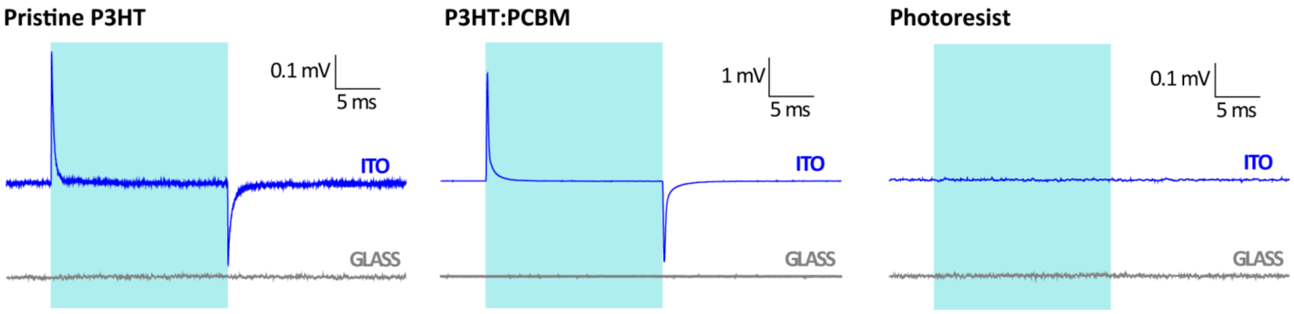

Figure S1: local photo-potentials at the polymer-electrolyte interface. Surface electrical potential are measured with the patch-clamp setup by micro-manipulating the pipette in the close proximity (~ 2 μm) of the polymer-electrolyte interface. Both the pipette and the bath are filled with extracellular solution (see Methods) to avoid junction potentials artefacts. Recordings are performed in voltage-clamp mode, setting the measured current to zero under dark conditions; surface potential is then calculated by multiplying the current trace measured upon illumination by the pipette resistance. The electrically active materials (P3HT and P3HT:PCBM) show a transient potential at the light switching on and off. This transient is ascribed to the formation of a photopotential due to electron injection from the active material into the ITO layer. The resulting current gives a capacitive charging of the interface with the electrolyte, producing a transient potential in the bath in close proximity to the polymer. Due to increased efficiency in charge generation and separation in the P3HT:PCBM blend, the signal is about one order of magnitude higher with respect to the pristine P3HT sample. The ITO layer is essential for the instauration of the photo-potential, since it acts as electron collector from the active material, enabling charges to be spatially separated before they can recombine. In the case of bare glass substrates, indeed, no surface potential is measured. Finally, for the photoresist on both ITO and ITO-less substrates, no signal is recorded, due to the absence of charge generation in the material.

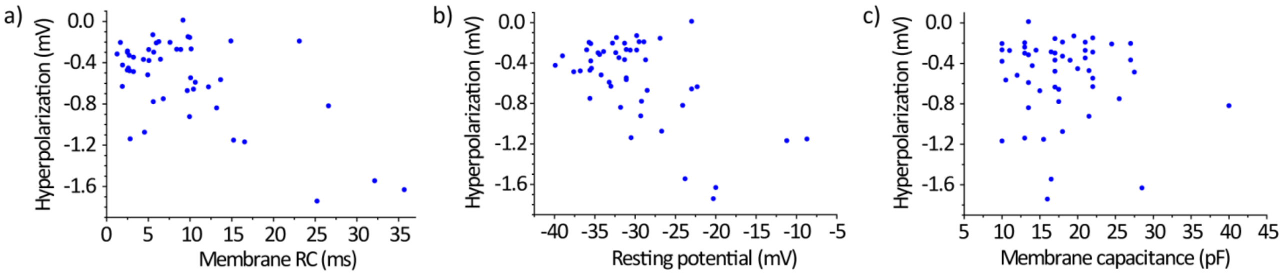

Figure S2: hyperpolarisation does not correlate with electrical cell parameters. Scatter plots of the hyperpolarisation hyp_{on} measured just before the end of a 200 ms light pulse (intensity 57 mW/mm^2) against different electrical properties of the cell membrane. At variance with the cell membrane depolarization, no evident correlation can be inferred from the above data, possibly indicating the different origin of the two signals.

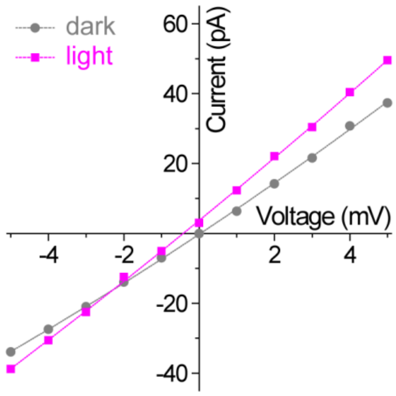

Figure S3: electrical properties of cell membrane near resting potential in dark and in light. The effect of photostimulation on the membrane electrical properties is studied by analysing the cell I-V characteristics for potentials close to the resting membrane potential (RMP). I-V characteristics in dark (grey line and symbols) and light (magenta line and symbols) were extracted from the measurements described in the main text (Fig. 4d). The experimental data (circles and rectangles) are then fitted with a second order polynomial (solid lines). The resting potential in light *versus* dark was extracted from the intercept of the curves with the x-axis, while the membrane resistance in the two cases was calculated as the inverse of the curves slopes in those points.

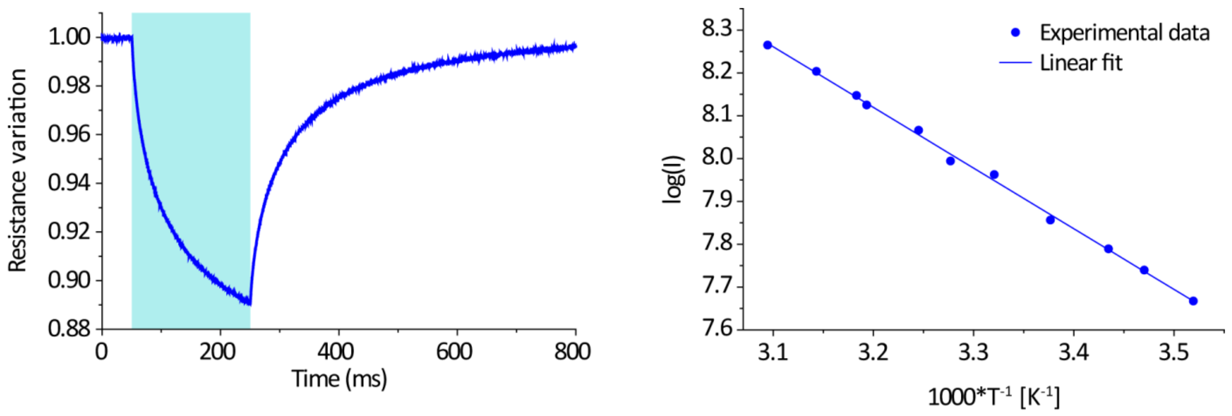

Figure S4: calibrated pipette measurements of local temperature. Temperature increase of the bath at the polymer interface is measured with the method of the calibrated pipette [8]. A patch pipette is micromanipulated in close proximity (~ 2 μm) of the active material, with both the pipette and the bath filled with a 0.2 M NaCl aqueous solution. Due to the temperature increase in the bath upon illumination of the active material, the resistance of the pipette decreases (left panel, for a 200 ms pulse at 57 mW/mm^2). To extract the actual temperature, the pipette resistance is pre-calibrated by measuring the variation in pipette current by controlling the bath temperature. In the right panel, the Arrhenius plot of the pipette current for temperatures in the 10 °C – 50 °C range is shown, from which an activation energy of E_a = 2.79 kcal/mol is extracted.

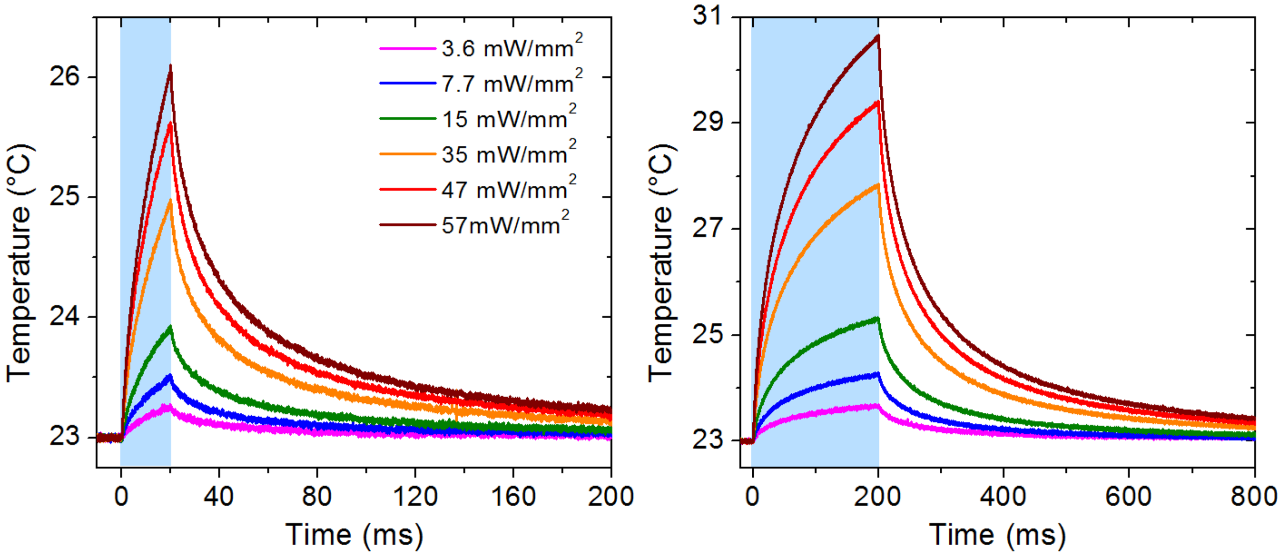

Figure S5: local temperature measurements on photoresist. Local temperature traces measured in close proximity of the substrate upon illumination at increasing light intensities for a photoresist sample (on glass). The traces have been recorded both for 20 ms pulses (left) and for 200 ms ones (right), with a LED light source at λ = 435 nm. The traces closely resemble the ones measured on the P3HT sample, in accordance with a similar light absorption and comparable thermal properties of the substrates.

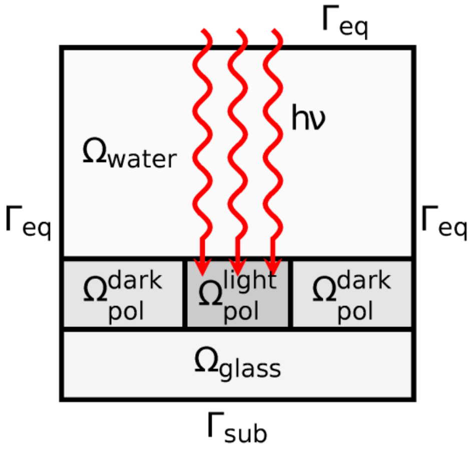

Figure S6: schematic representation of a section of the computational domain for thermal simulations. With Ω_{water}, Ω_{pol} and Ω_{glass} we denote the regions occupied by the solution, the polymer and the glass substrate, respectively. Ω_{pol} comprises an illuminated part Ω_{pol}^{light} where heat is generated, and a dark part Ω_{pol}^{dark}. The boundary Γ_{sub} represents the contact area with the plastic substrate where null flux conditions are applied, while Γ_{eq} denotes regions far enough from the absorption region to be considered unperturbed. The scheme is not in scale.

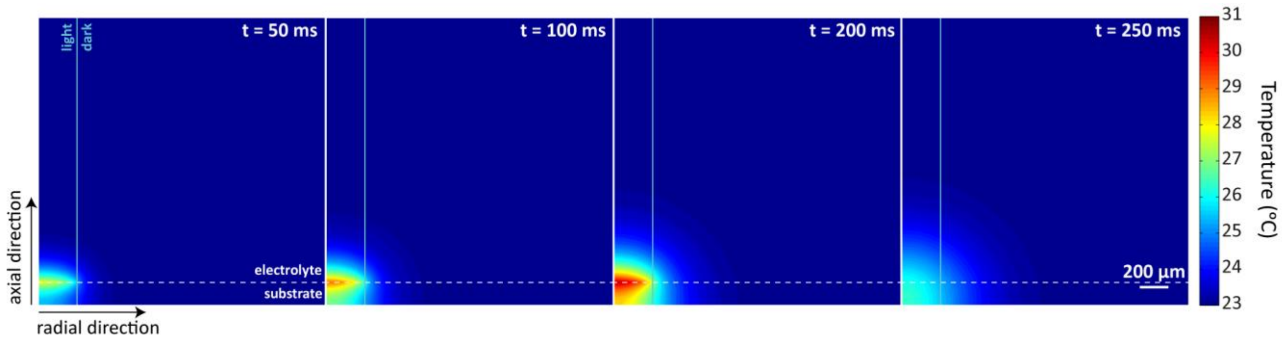

Figure S7: spatial distribution of the temperature profile during illumination. The four panels represent the distribution in space (along the radial and axial direction, given the cylindrical symmetry of the system) of the local temperature at different times during and after a 200 ms pulse (switched on at t = 0 ms) of 57 mW/mm^2, as obtained from the numerical simulation described in the supplementary discussion. The light beam has a dimension of about 270 µm at the focal point on the substrate. It can be seen that the temperature increase is laterally confined to the illuminated region, while the bulk of the solution remains at the baseline temperature during the pulse; in the axial direction the temperature quickly drops within the first few hundreds of micrometers from the substrate.

Supplementary Reference

[1] Patrício, P. & Calado, H. Correlation between thermal, optical and morphological properties of heterogeneous blends of poly (3-hexylthiophene) and thermoplastic polyurethane. *J. Phys.: Condens. Matter* **18**, 7529-7542 (2006).

[2] Van Bavel, S. S., Sourty, E., de With, G. & Loos, J. Controlling The 3D Nanoscale Organization Of Bulk Heterojunction Polymer Solar Cells. *Chin. J. Polym. Sci.* **27**, 1 (2009).

[3] Duda, J. C., Hopkins, P. E., Shen, Y. & Gupta, M. C. Thermal transport in organic semiconducting polymers. *Appl. Phys. Lett.* **102**, 251912 (2013).

[4] Gatti, E., Micheletti, S. & Sacco, R. A new Galerkin framework for the drift-diffusion equation in semiconductors. *East-West J. Numer. Math.* **6**, 101-136 (1998).

[5] Hindmarsh, A. C. *et al.* SUNDIALS: Suite of Nonlinear and Differential/Algebraic Equation Solvers. *ACM T. Math. Soft.*, **31**, 363-396 (2005).

[6] Eaton, J. W. *et al.* GNU Octave version 3.0.1 manual: a high-level interactive language for numerical computations. CreateSpace Independent Publishing Platform (2009).

[7] Shapiro, M. G. *et al.* Infrared light excites cells by changing their electrical capacitance. *Nat. Comm.* **3**, 736 (2012).

[8] Yao, J., Liu, B. & Qin, F. Rapid temperature jump by infrared diode laser irradiation for patch-clamp studies. *Biophys. J.* **96**, 3611-3619 (2009).
